# Supplementary material for: Multiple sources of volunteered geographic information strengthen holistic estimates of lake visitation
Source: PLoS One. 2026 Feb 2;21(2):e0341808. doi: 10.1371/journal.pone.0341808 (PMC12863485; doi:10.1371/journal.pone.0341808)
Supplement: Attachment — Submitted filename: PLOS_ReviewResponse.docx [file pone.0341808.s001.docx]

Reviewer #1:

*We thank the reviewer for their thoughtful questions, which have prompted edits that ultimately strengthen our manuscript.*

1.How were the five VGI sources (Flickr, eBird, iNaturalist, Twitter, and Gaia GPS) selected, and do they comprehensively represent the full range of lake users?

*We primarily studied these sources because they are the most readily available and most commonly researched by previous studies (factors which may be related, we acknowledge). We have clarified this in the text (lines 148-152): "These five platforms were selected largely because the data are accessible to researchers (e.g., through public acquisition interfaces or data sharing agreements, described below). Four of the platforms have also been the primary focus of previous studies of competing methods for estimating visitation (see reviews by Ghermandi et al., Wilkins et al.) [21, 61]."*

*Given the Reviewer’s question, we decided to add discussion (lines 349-361, quoted below), of the challenges to understanding issues of bias and representation in these datasets. The datasets are not comprehensive nor are they representative of all lake users, as has been discussed at length in previous studies (now cited). The purpose of our study is to quantify and therefore understand the representativeness of these datasets – through their association with actual visitation – and how representativeness differs among platforms,*

*We clarify this in the following paragraph: "What remains a persistent challenge is understanding how the use of different mobile device applications, and therefore the volumes of VGI data from different platforms, are related to specific CES for people. Havinga et al. [37] proposed a framework of CES service categories (e.g., activity, aesthetic, artistic, knowledge) and how each is associated with specific types of VGI. According to the framework, Gaia GPS is an indicator of activity services because its users are physically interacting with the environment, whereas Flickr is an indicator of aesthetic appreciation or artistic inspiration, and eBird an indicator of connection to nature. However, there is also growing recognition that CES benefits may vary considerably within the user-group of a single VGI source [48]. VGI sources probably represent more than just one CES, and some CES are easier to recognize and classify through VGI analysis than others. Understanding representation of specific types of CES, or lack thereof, in selected data sources and across different ecosystems is critical for considering how ecosystem valuation using VGI analyses can support natural resource management and planning."*

2.What measures were taken to mitigate potential biases in VGI data, such as user demographics, regional preferences, or varying levels of social media engagement?

*We appreciate the question, and note these concerns are typically raised when it comes to VGI data. First, it is important to recognize that data from the VGI sources used in our study (and most studies) are purposely anonymous, and thus user demographics are unknown. We discuss this limitation in the manuscript here (lines 449-452): "Moreover, given that VGI based approaches may poorly represent specific sociodemographic groups in society holistic representation of lake users in CES assessments may be best achieved through a combination of VGI, other digital surveys, and in-person workshops [58]." Given this anonymity, regional preferences in VGI sources are also largely unquantifiable.*

*Varying social media engagement is definitely something that we carefully considered throughout the study. To address any potential biases we decided to condense VGI records to user-days, as described in the text here (lines 173-179): "We then aggregated posts by VGI source, user ID, lake, and day to calculate the number of user-days – or distinct users who visit per day – per VGI source at each lake [23]. In other words, if a user posted to the same platform from the same lake multiple times in one day this is considered a single lake user-day. This aggregation is done to prevent users who post to a VGI source multiple times successively from a single location (i.e., high engagement) from dominating the analysis in comparison to users who post less frequently, but nevertheless are visiting lakes." Otherwise, our research is exploring whether and to what extent the biased convenience sample is related with on-site estimates of visitation.*

3.The study states that all VGI sources contributed significantly to the top-performing visitation models (R² = 0.85). Were alternative modeling approaches considered, and how did they compare in terms of predictive accuracy?

*An objective of our study was to compare relative contributions of different VGI sources, not to compare different modeling frameworks. To this end, we compared models informed by different combinations of VGI sources (Table 1), and found that the model informed by all five VGI sources performed similarly based on R^2^ and RMSE to other top models incorporating fewer VGI sources (lines 301-306): "We used the model informed by all five VGI datasets (n = 231, combined R^2^ = 0.85, in-sample testing) to predict empirical visitation across all sites (and for years in which we lacked empirical data for certain lakes). Predicted mean annual visitation positively correlated with on-site visitation, though this model and the others we tested explained only a modest percentage of the data’s variance in out-of-sample testing (Delta AICc = 4.62, RMSE = 1.06, R^2^ = 0.12) (Figure 5)."*

4.How was the robustness of the models tested? Were cross-validation techniques or out-of-sample tests used to ensure reliability?

*We are unsure if the Reviewer missed our description of cross-validation in the original submission, or if our description was unclear. If the latter, we invite the Reviewer to follow-up with specific suggestions on how to make the text clearer, if it is not. We used out-of-sample tests to assess model generalizability across predictions (lines 207-217): "Next, we evaluated the top visitation models’ ability to predict visitation at lakes lacking on-site data with out-of-sample testing. For each top model, we trained the model on all observations from two-thirds of the lakes in our dataset and tested it over all observations for the remaining one-third of lakes, repeated for 1,000 estimates. Given discrepancies in the number of total years each lake was sampled over the five years of our study (e.g., some lakes were only sampled for two or three years while others were sampled for all five), we grouped cross-validation by lake to ensure accurate representation. In other words, all years of data from a single lake were included in either the training or test dataset. This approach also allowed us to assess how effective a model trained on one set of lakes was at predicting visitation on an entirely different set of lakes. We calculated the average root mean squared error (RMSE) and R-squared of the out-of-sample tests to assess the models."*

5.The study mentions that Twitter and Gaia GPS were more robust predictors than eBird and iNaturalist. Is there a threshold at which adding additional VGI sources no longer improves the model?

*As shown in Table 1, Twitter and Gaia GPS were the two VGI sources in the top-performing visitation model when evaluated by AIC, but all five VGI sources are included in models within Delta AICc < 2. Inherently, AIC rankings will be biased toward models with fewer variables – or fewer VGI sources. Based on these results we proceeded with the model informed by all five VGI sources, which performed similarly based on R^2^ and RMSE to other top models incorporating fewer VGI sources (Table 1). One could presume that adding additional VGI sources beyond Twitter and Gaia GPS no longer improves the model based solely on statistical performance (AIC) – but as we describe in the manuscript different VGI sources represent different lake user-groups, and thus by including a broader array of VGI sources our estimates incorporate a greater diversity of lake users.*

6.Did the study analyze whether different seasons or specific events affected the utility of various VGI sources?

*We certainly wanted to analyze if seasonality or discrete events affected the utility of different VGI sources. Unfortunately, we were unable to do so given the temporal extent of empirical data as described in the text here (lines 183-185): "Volunteers (typically lakeside residents) counted the number of boaters, swimmers, and other recreationists on the lake water and shoreline at instantaneous points in time, bi-weekly from May through October, over the years 2015 to 2019." Thus, we do not have empirical data to model VGI sources against for half of the year to examine seasonal effects, and we also lack the empirical data density needed over time to assess the influence of specific events.*

7.Given that built lakeside infrastructure was the primary driver of visitation, what specific infrastructure improvements could be recommended based on the findings?

*We describe the tradeoffs managers face in facilitating competing lake use types here, and recommend spatial zoning to accommodate lake uses that correlate with increased visitation as well as less social lake pursuits (lines 386-401): "Managers face a challenge in determining how to balance demands for competing CES through lakeside development or restoration. While water-based activities contribute to human well-being, they can simultaneously impose stressors on aquatic systems such as depleting aquatic and riparian habitat quality, altering species’ behaviors, and changing the biogeochemical cycles of aquatic ecosystems [52, 53]. Negative impacts to the natural environment subsequently adversely affect nature-based activities such as birdwatching and aesthetic appreciation. These competing demands are somewhat addressed by the heterogeneous spatial distribution of public activity-specific infrastructure (e.g., boat ramps, fishing docks, swimming beaches, natural preserves) at lakes, but access to lakeside environments supporting different lake-based CES is not equitable across the landscape. When allocating funds and resources to lakeside enhancement projects, managers should carefully consider tradeoffs between enriching built environments and introducing environmental stressors related to lake use hotspots (e.g., garbage, deteriorating riparian quality) which may impair CES derived from more natural lake environments [54]. Furthermore, intentional spatial zoning of public lake shorelines can help facilitate multiple types of CES that may directly conflict with one another, such as swimming and fishing [55]."*

8. It is suggested to add articles entitled “Hassan et al. Effect of Climate Change on Wetland Areas in West Iraq Using Satellite Data and GIS Techniques”, “Rattanarat et al. Government Policy Influence on Land Use and Land Cover Changes: A 30-Year Analysis” and “Setiowati & Koestoer. Valuation of Urban Green Open Spaces Using the Life Satisfaction Approach” to the literature review.

*We considered these sources in our revisional literature review, but ultimately concluded that their subject areas of focus and methodologies (land use change detection with remote sensing and urban park planning) were not nearly relevant enough to include in our manuscript, which is already at the citation limit.*

9. How can policymakers ensure equitable access to lakes while considering the digital divide (i.e., certain socioeconomic groups may be underrepresented in VGI data due to lower smartphone or social media use)?

*We suggest that policymakers leverage VGI data in tandem with other in-person and digital surveying approaches here (lines 449-452): "Moreover, given that VGI based approaches may poorly represent specific sociodemographic groups in society holistic representation of lake users in CES assessments may be best achieved through a combination of VGI, other digital surveys, and in-person workshops [60]."*

10.The study notes that VGI is not a substitute for on-site visitor data. How can future studies effectively integrate VGI with empirical on-site observations for more accurate estimates?

*We recommend that future studies aim to directly relate different VGI sources to on-site lake users by directly surviving in-person visitors about their VGI contributions and associated CES here (lines 434-452): "Future research should explicitly show how specific sources of VGI are associated with CES through on-site surveys that categorize lake visitors by activity type and the value they receive from the visit. Previous work has demonstrated discrepancies between the CES described in visitor use survey responses and CES inferred from automated analysis of text and images included in VGI, suggesting that further research is needed to understand how well VGI reflects visitors' perceived benefits at recreational sites of interest [58]. Here we argue that multiple sources of VGI better estimate visitation because more diverse user-groups are represented, but this is likely an oversimplification of the true CES the individuals that posted to mobile device applications are experiencing. Urban and suburban lakes offer numerous benefits to humans that may not be fully reflected in any source of VGI, such as improving health, reducing stress, providing social and place-based belonging, and mediating negative impacts of urban heat and noise [3, 59]. A comprehensive in-person survey asking lake users which CES they relate to through their VGI posts and which benefits they feel are not captured by their mobile device application activity would further refine the capabilities and limitations of VGI for estimating lake-based CES. Moreover, given that VGI based approaches may poorly represent specific socio-demographic groups of people, holistic representation of lake users in CES assessments may be best achieved through a combination of measurement techniques including VGI, surveys, and in-person workshops [60]."*

11.Were there any limitations in data availability or quality that affected the study’s conclusions, and how might future research address them?

*We addressed this question in the original submission, which we have now bolstered with additional information. Here is the text (lines 459-471): "* *There are practical and ever-evolving limitations to VGI acquisition. Notably, geolocated Instagram posts are no longer available through an API, and since the completion of our analyses Twitter (now X) has placed burdensome costs and rate limits and on its academic API. Furthermore, mobile device application use and the popularity of individual applications changes over time, so VGI sources may not consistently reflect on-site activities [32]. Given the increasing hurdles raised by VGI companies to access these data and the temporary nature of use trends in mobile device application activity [59], practitioners should consider the relative return on investment of acquiring many different VGI sources. Future research could explicitly calculate the utility cost (i.e., necessary personnel and monetary investment) of different VGI sources relative to empirical data collection methods. In light of the uncertainty surrounding long-term research access to some platforms (e.g., Twitter) and VGI’s ability to simply enhance but not replace on-site estimates, collection of on-site visitation and survey data remains critical for recreational site management [28]."*

Reviewer #2:

The manuscript “Multiple sources of volunteered geographic information strengthen holistic estimates of lake visitation” presents an interesting approach to estimating lake visitation using multiple volunteered geographic information (VGI) sources. While the topic is relevant and timely, there are significant methodological, conceptual, and presentation issues that prevent it from being suitable for publication in its current form.

*We thank the reviewer for their careful consideration of our manuscript and noting that it is both relevant and timely. Here, we have endeavored to address their concerns through significant text edits and justification of our approach, methods, and conclusions.*

1. Methodological Limitations

The study relies heavily on volunteered geographic information (VGI) without sufficiently addressing the potential biases and limitations associated with such data sources.

*Based on the reviewer's comments it is clear we did not adequately articulate the potential biases of VGI sufficiently. We welcome this opportunity to further elaborate on additional limitations, and have added an additional paragraph on known limitations and accompanying references to our discussion section titled "Limitations and future directions" here (lines 423-436): "The VGI data sources used in this study are all convenience samples that are not representative of the population of lake users [23, 30, 31]. Visitors represented by some VGI sources, for example, are known to be younger [31] or biased toward a particular gender [30] compared with actual visitors. Furthermore, there are reasons why lake visitation may be challenging to measure using VGI, compared with studies investigating potential of VGI for terrestrial parks and protected areas. Individuals participating in water-based activities – such as fishing, boating, and swimming – may be less likely to engage with their mobile device while visiting a waterbody [23]. This may result in lower amounts of available VGI and, since VGI patterns tend to more accurately represent empirical visitation at sites with greater volumes of VGI [31], the need to aggregate data to annual or monthly scales, as in this study. It remains unclear what the utility of VGI will be for estimating lake visitation over shorter timescales [28]. Future research should explicitly show how specific sources of VGI are associated with CES through on-site surveys that categorize lake visitors by activity type and the value they receive from the visit."*

There is no validation mechanism for comparing VGI-based estimates with actual visitor data beyond limited empirical counts.

*Yes, the reviewer is correct in stating even empirical data has limitations – trail counters, drone counts, and vehicle counts all estimate visitation at snapshots in time or over limited periods of deployment. Furthermore, all of these mechanisms for estimating visitor use are prone to their own sources of error. However, our study is no different from previous research sharing the same challenge. We have now added text to the new "Limitations and future directions" section to make this more explicit (lines 423-436)“The VGI data sources used in this study are all convenience samples that are not representative of the population of lake users [23, 30, 31]. Visitors represented by some VGI sources, for example, are known to be younger [31] or biased toward a particular gender [30] compared with actual visitors. Furthermore, there are reasons why lake visitation may be challenging to measure using VGI, compared with studies investigating potential of VGI for terrestrial parks and protected areas. Individuals participating in water-based activities – such as fishing, boating, and swimming – may be less likely to engage with their mobile device while visiting a waterbody [23]. This may result in lower amounts of available VGI and, since VGI patterns tend to more accurately represent empirical visitation at sites with greater volumes of VGI [31], the need to aggregate data to annual or monthly scales, as in this study. It remains unclear what the utility of VGI will be for estimating lake visitation over shorter timescales [28]. Future research should explicitly show how specific sources of VGI are associated with CES through on-site surveys that categorize lake visitors by activity type and the value they receive from the visit."*

The selection of VGI platforms (Twitter, Flickr, etc.) is not well-justified in terms of why they represent lake visitation more accurately than other available sources.

*When selecting different VGI platforms we navigated tradeoffs between large, geographically dispersed data, publicly and easily available datasets, and potential platforms being used based on common lake activities. If we want to use VGI platforms as valid indicators of visitation they need to be easily and readily available – managers do not have the luxury of spending years acquiring data that is not publically available. To be clear, we do not imply that VGI platforms broadly represent lake visitation more accurately than empirical approaches; this was not stated in the original submission. Rather, VGI platforms facilitate estimation of visitation at spatiotemporal scales that are impossible for managers to collect empirical data at given limited funding and personnel. This fact is now made clear here (lines 144-152) "When selecting different VGI platforms we navigated tradeoffs between large, geographically dispersed data, publicly and easily available datasets, and potential platforms being used based on common lake activities. If we want to use VGI platforms as valid indicators of visitation they need to be easily and readily available – managers do not have the luxury of spending years acquiring data that is not publicly available. These five platforms were selected largely because the data are accessible to researchers (e.g., through public acquisition interfaces or data sharing agreements, described below). Four of the platforms have also been the primary focus of previous studies of competing methods for estimating visitation (see reviews by Ghermandi et al., Wilkins et al.) [21, 61]."*

2. Inconsistencies in Data Analysis

The statistical models lack clear justification for why certain variables were chosen over others.

*This is a valid point. While there are limitless variables that we could have incorporated into the revealed preference model, we specifically chose to focus on parameters (water quality, tree cover, infrastructure availability) that could be influenced by lake and park management. We have edited our justification for why certain variables were chosen over others here (lines 233-236): "These predictors were selected because they can reasonably be influenced by natural resource managers, and previous studies have demonstrated visitors are willing to travel further to lakes and parks which offer superior water quality and built and natural amenities [24, 25]."*

There is insufficient discussion on potential data gaps and representativeness issues, particularly in lakes where VGI activity is low or absent.

*We have addressed this comment by adding to the discussion here (lines 428-432): "Individuals participating in water-based activities – such as fishing, boating, and swimming – may be less likely to engage with their mobile device while visiting a waterbody [23]. This may result in lower amounts of available VGI and, since VGI patterns tend to more accurately represent empirical visitation at sites with greater volumes of VGI [31], the need to aggregate data to annual or monthly scales, as in this study."*

The cross-validation approach is unclear and does not convincingly demonstrate the generalizability of the results.

*We don’t entirely understand this comment. We have added sentences to clarify our cross-validation approach here (lines 207-217): "Next, we evaluated the top visitation models’ ability to predict visitation at lakes lacking on-site data with out-of-sample testing. For each top model, we trained the model on all observations from two-thirds of the lakes in our dataset and tested it over all observations for the remaining one-third of lakes, repeated for 1,000 estimates. Given discrepancies in the number of total years each lake was sampled over the five years of our study (e.g., some lakes were only sampled for two or three years while others were sampled for all five), we grouped cross-validation by lake to ensure accurate representation. In other words, all years of data from a single lake were included in either the training or test dataset. This approach also allowed us to assess how effective a model trained on one set of lakes was at predicting visitation on an entirely different set of lakes. We calculated the average root mean squared error (RMSE) and R-squared of the out-of-sample tests to assess the models." Cross-validation is known to be an unbiased test of model generalizability across predictions (Olden, J.D. and D.A. Jackson. 2000. Torturing the data for the sake of generality: How valid are our regression models? Ecoscience 7:501-510.; and many other papers).*

3. Theoretical and Conceptual Weaknesses

The manuscript does not engage deeply with existing literature on ecosystem services and lake recreation studies.

*We appreciate this comment and the opportunities to engage more with the literature. We have added the following citations in our revisions:*

1. *Allan JD, Smith SD, McIntyre PB, Joseph CA, Dickinson CE, Marino AL, Biel RG, Olson JC, Doran PJ, Rutherford ES, Adkins JE. Using cultural ecosystem services to inform restoration priorities in the Laurentian Great Lakes. Front Ecology Environ. 2015;13(8): 418-24. doi:* [*10.1890/140328*](https://doi.org/10.1890/140328)

*Lines 401-404: "When allocating funds and resources to lakeside enhancement projects, managers should carefully consider tradeoffs between enriching built environments and introducing environmental stressors related to lake use hotspots (e.g., garbage, deteriorating riparian quality) which may impair CES derived from more natural lake environments [54]."*

2. *Meyerhoff J, Klefoth T, Arlinghaus R. The value artificial lake ecosystems provide to recreational anglers: Implications for management of biodiversity and outdoor recreation. J Environ Manage. 2019;252: 109580. doi:* [*10.1016/j.jenvman.2019.109580*](https://doi.org/10.1016/j.jenvman.2019.109580)

*Lines 405-406: "Furthermore, intentional spatial zoning of public lake shorelines can help facilitate multiple types of CES that may directly conflict with one another, such as swimming and fishing [55]." Meyerhoff et al. 2019*

There is limited discussion on socio-economic factors that might influence visitation patterns beyond infrastructure availability.

*We concur that our study offers a limited discussion on socio-economic factors, and this phrasing extends the reach of our work beyond reasonable conclusions. We have removed the limited mentions of this in the text.*

The study fails to acknowledge alternative data collection methods (e.g., sensor-based visitation tracking, surveys, or observational studies).

*We discuss alternative data collection methods in the original submission here (lines 66-70): "Human activity on waterbodies across time and space is frequently inferred from sparsely conducted visitor counts providing data with limited spatiotemporal scope [14]. Conventional approaches typically rely on direct in-person surveys or passive sensors for counting people or vehicles (often at boat launches), while other studies rely on mail-in questionnaires sent to registered anglers or boaters [15]."*

4. Writing and Structure Issues

The manuscript is too descriptive and lacks a strong analytical depth in interpreting the results.

*We did our best to navigate the additional descriptive nature that the reviewer requested in comments above juxtaposed with the suggestion that the text is too descriptive.*

Some sections are redundant, particularly in the introduction and discussion.

*We removed the sentences "Data from VGI sources is believed to provide more robust measures of visitation because it encompasses a broader cross-section of the public who may be traveling for different reasons," and "Here, we present the first VGI-informed lake visitation models with data from multiple VGI sources," from paragraph four in the introduction to reduce some redundancy.*

The figures and tables need better integration with the text, and some results are not fully explained.

*We appreciate the comment, but admittedly found it challenging to make large changes since specific issues were not listed by the reviewer. Nevertheless, in the revision we worked hard to ensure that all results are fully explained and figures are fully integrated with the text. Additional friendly reviews from colleagues also ensured that any issues have been remedied.*
