# Supplementary material for: Multiple sources of volunteered geographic information strengthen holistic estimates of lake visitation
Source: PLoS One. 2026 Feb 2;21(2):e0341808. doi: 10.1371/journal.pone.0341808 (PMC12863485; doi:10.1371/journal.pone.0341808)
Supplement: Attachment — Submitted filename: ReviewerResponse.docx [file pone.0341808.s002.docx]

Journal Requirements:

When submitting your revision, we need you to address these additional requirements.

1. Please ensure that your manuscript meets PLOS ONE's style requirements, including those for file naming. The PLOS ONE style templates can be found at

<https://journals.plos.org/plosone/s/file?id=wjVg/PLOSOne_formatting_sample_main_body.pdf> and

[https://journals.plos.org/plosone/s/file?id=ba62/PLOSOne_formatting_sample_title_authors_affiliations.pdf](https://track.editorialmanager.com/CL0/https:%2F%2Fjournals.plos.org%2Fplosone%2Fs%2Ffile%3Fid=ba62%2FPLOSOne_formatting_sample_title_authors_affiliations.pdf/1/010f0199b8694eb4-b21e8ffe-3faf-4834-bd99-0d4221415b52-000000/j22oum9UHkQMFCS8VqSZ79nvYPaBvJKIAdWJQ3JFNAs=231).

*We have edited file names and document formatting to meet these requirements.*

2. In your Methods section, please include additional information about your dataset and ensure that you have included a statement specifying whether the collection and analysis method complied with the terms and conditions for the source of the data.

*We have added the following statement to our Methods and are happy to provide additional information about the dataset with specific guidance: “All VGI data collection and analysis methods complied with the terms and conditions for each respective data source.” (l. 208-209)*

3. Please note that PLOS One has specific guidelines on code sharing for submissions in which author-generated code underpins the findings in the manuscript. In these cases, we expect all author-generated code to be made available without restrictions upon publication of the work. Please review our guidelines at <https://journals.plos.org/plosone/s/materials-and-software-sharing#loc-sharing-code> and ensure that your code is shared in a way that follows best practice and facilitates reproducibility and reuse.

*We have made our code and the data we are permitted to share available on Zenodo (link:* [*10.5281/zenodo.17654557*](https://doi.org/10.5281/zenodo.17654557)*).*

4. Thank you for stating in your Funding Statement:

“This work was supported by a U.S. Geological Survey Northwest Climate Adaptation Science Center award G17AC00218 to Rachel Fricke, the Future Rivers program at the University of Washington as part of a NSF National Research Traineeship award (DGE 1922004), and the University of Washington eScience Institute.”

Please provide an amended statement that declares *all* the funding or sources of support (whether external or internal to your organization) received during this study, as detailed online in our guide for authors at <http://journals.plos.org/plosone/s/submit-now>.  Please also include the statement “There was no additional external funding received for this study.” in your updated Funding Statement.

Please include your amended Funding Statement within your cover letter. We will change the online submission form on your behalf.

*We have provided an amended Funding Statement in the cover letter.*

5. We note that you have indicated that there are restrictions to data sharing for this study. PLOS only allows data to be available upon request if there are legal or ethical restrictions on sharing data publicly. For more information on unacceptable data access restrictions, please see <http://journals.plos.org/plosone/s/data-availability#loc-unacceptable-data-access-restrictions>.

Before we proceed with your manuscript, please address the following prompts:

a) If there are ethical or legal restrictions on sharing a de-identified data set, please explain them in detail (e.g., data contain potentially identifying or sensitive patient information, data are owned by a third-party organization, etc.) and who has imposed them (e.g., a Research Ethics Committee or Institutional Review Board, etc.). Please also provide contact information for a data access committee, ethics committee, or other institutional body to which data requests may be sent.

b) If there are no restrictions, please upload the minimal anonymized data set necessary to replicate your study findings to a stable, public repository and provide us with the relevant URLs, DOIs, or accession numbers. For a list of recommended repositories, please see

<https://journals.plos.org/plosone/s/recommended-repositories>. You also have the option of uploading the data as Supporting Information files, but we would recommend depositing data directly to a data repository if possible.

We will update your Data Availability statement on your behalf to reflect the information you provide.

*We have edited our data availability statement and provide a revised version below indicating that all shareable datasets are now available on Zenodo:*

*“All shareable data underlying the results presented in this study are available on Zenodo [*[*10.5281/zenodo.17654557*](https://doi.org/10.5281/zenodo.17654557)*]. VGI data cannot be shared publicly because the data are owned by third parties and our terms of use do not allow us to share the data. VGI data related to this study, including volunteered geographic information records from Flickr, Twitter (now X), Gaia GPS, eBird, and iNaturalist, are available upon download or request from the following webpages and application programming interfaces (APIs): Flickr [*[*https://www.flickr.com/services/developer/api/*](https://www.flickr.com/services/developer/api/)*], eBird [*[*https://science.ebird.org/en/use-ebird-data/download-ebird-data-products*](https://science.ebird.org/en/use-ebird-data/download-ebird-data-products)*], iNaturalist [doi:* [*10.15468/ab3s5x*](https://doi.org/10.15468/ab3s5x)*], and Twitter (X) [*[*https://developer.x.com/en/docs/x-api*](https://developer.x.com/en/docs/x-api)*]. Please note that the free Academic API the authors used to acquire Twitter (X) data is now deprecated under new company policies. Researchers interested in accessing Gaia GPS data must submit a formal data access request directly to the company outlining their research goals and ensuring compliance with relevant privacy regulations [*[*https://www.gaiagps.com/*](https://www.gaiagps.com/)*].”*

6. We note that Figures 1 & 2 in your submission contain [map/satellite] images which may be copyrighted. All PLOS content is published under the Creative Commons Attribution License (CC BY 4.0), which means that the manuscript, images, and Supporting Information files will be freely available online, and any third party is permitted to access, download, copy, distribute, and use these materials in any way, even commercially, with proper attribution. For these reasons, we cannot publish previously copyrighted maps or satellite images created using proprietary data, such as Google software (Google Maps, Street View, and Earth). For more information, see our copyright guidelines: <http://journals.plos.org/plosone/s/licenses-and-copyright>.

We require you to either (1) present written permission from the copyright holder to publish these figures specifically under the CC BY 4.0 license, or (2) remove the figures from your submission:

a. You may seek permission from the original copyright holder of Figures 1 & 2 to publish the content specifically under the CC BY 4.0 license.

We recommend that you contact the original copyright holder with the Content Permission Form (<http://journals.plos.org/plosone/s/file?id=7c09/content-permission-form.pdf>) and the following text:

“I request permission for the open-access journal PLOS ONE to publish XXX under the Creative Commons Attribution License (CCAL) CC BY 4.0 (<http://creativecommons.org/licenses/by/4.0/>). Please be aware that this license allows unrestricted use and distribution, even commercially, by third parties. Please reply and provide explicit written permission to publish XXX under a CC BY license and complete the attached form.”

Please upload the completed Content Permission Form or other proof of granted permissions as an "Other" file with your submission.

In the figure caption of the copyrighted figure, please include the following text: “Reprinted from [ref] under a CC BY license, with permission from [name of publisher], original copyright [original copyright year].”

 b. If you are unable to obtain permission from the original copyright holder to publish these figures under the CC BY 4.0 license or if the copyright holder’s requirements are incompatible with the CC BY 4.0 license, please either i) remove the figure or ii) supply a replacement figure that complies with the CC BY 4.0 license. Please check copyright information on all replacement figures and update the figure caption with source information. If applicable, please specify in the figure caption text when a figure is similar but not identical to the original image and is therefore for illustrative purposes only.

The following resources for replacing copyrighted map figures may be helpful:

USGS National Map Viewer (public domain): <http://viewer.nationalmap.gov/viewer/>

The Gateway to Astronaut Photography of Earth (public domain): <http://eol.jsc.nasa.gov/sseop/clickmap/>

Maps at the CIA (public domain): <https://www.cia.gov/library/publications/the-world-factbook/index.html> and <https://www.cia.gov/library/publications/cia-maps-publications/index.html>

NASA Earth Observatory (public domain): <http://earthobservatory.nasa.gov/>

Landsat: <http://landsat.visibleearth.nasa.gov/>

USGS EROS (Earth Resources Observatory and Science (EROS) Center) (public domain): [http://eros.usgs.gov/#](http://eros.usgs.gov/)

Natural Earth (public domain): <http://www.naturalearthdata.com/>

If the reviewer comments include a recommendation to cite specific previously published works, please review and evaluate these publications to determine whether they are relevant and should be cited. There is no requirement to cite these works unless the editor has indicated otherwise.

*We have edited Figures 1 and 2 to use freely available US Census Bureau basemaps instead, given GADM’s limitations on dissemination for commercial use.*

*We have also updated the figure captions:*

***“Fig 1.*** *Locations of 50 lakes in Western Washington, United States. The basemap is freely available from the US Census Bureau.” (l. 157-158)*

***“Fig 2.*** *Map of cumulative VGI user-days at 50 lakes in Western Washington for 2015-2019 on eBird (dark blue), iNaturalist (teal), Flickr (yellow), Twitter (orange), and Gaia (red). Point size corresponds to the number of user-days and point locations are jittered to ease interpretation. The basemap is freely available from the US Census Bureau.” (l. 328-331)*

7. Please review your reference list to ensure that it is complete and correct. If you have cited papers that have been retracted, please include the rationale for doing so in the manuscript text, or remove these references and replace them with relevant current references. Any changes to the reference list should be mentioned in the rebuttal letter that accompanies your revised manuscript. If you need to cite a retracted article, indicate the article’s retracted status in the References list and also include a citation and full reference for the retraction notice.

*We have reviewed our reference list and formatting and attest that it is complete and correct. We do not cite any retracted articles.*

**Reviewer #1:**

I have read the revised version of the manuscript as well as the authors’ responses to the comments. In many cases, I am convinced by the revisions; however, I still have some additional comments that I believe could further improve the work.

*We thank the reviewer for their affirmation of our efforts to address previous reviewers’ comments and their suggestions to further strengthen our manuscript. We have endeavored to address all of their comments through our revisions described below.*

I found myself a bit confused about the model and the modeling process. Was the main aim only to explore the relationship between social media–recorded visits and actual on-site visitation? If so, what is the source of the real visitation data (on-site visitation), and is it publicly accessible? I also did not clearly understand what is meant by the “combined R².” The reported value of 0.85 is mentioned in the text but does not appear in Table 1.

*Yes, the main aim of our manuscript was to examine the relationship between visitation estimates derived from different combinations of VGI data sources and on-site visitation. On-site visitation data comes from instantaneous counts collected by county volunteers, as described in the “On-site Lake Visitation” subsection of the Methods. The data is available in our public Zenodo repository [*[*10.5281/zenodo.17654557*](https://doi.org/10.5281/zenodo.17654557)*]. “Combined R²” refers to the proportion of variance in on-site visitation that is explained by the all VGI sources model (e.g.,in-sample testing), whereas the R² values in Table 1 are from out-of-sample testing of candidate models (as described in the table caption). We have further clarified this by editing the abstract text, where in-sample was not previously specified: “All VGI sources were included in the top-performing visitation models, suggesting they provide significant and unique contributions to estimates of overall lake use (combined R^2^ = 0.85, in-sample testing)” (l. 35-37).*

It also seems that the relationship between visitation and its influencing factors has been modeled, with results suggesting that “built lakeside infrastructure” is the most important factor. However, the explanations regarding the modeling approach, data preparation, regression coefficients, significance tests, and similar details are either too brief or missing altogether.

*We apologize for not offering a sufficient description of our methods in the original submission. We have rectified this omission by editing the “Revealed Preference Model” subsection of the Methods to further clarify these details: “We modeled visitation estimates as a function of lake amenities, water quality, and shoreline tree cover to assess the associations between measures of lake attractiveness and degree of human use. Visitation estimates came from the previously described visitation model which predicted visitation from all VGI sources at all 50 lakes for the five years of our study, thus also including years during which we lacked on-site data at certain lakes. Correlated lake attributes related to the presence of amenities (parks, bathrooms, shelters, public docks, playgrounds, and swimming beaches) were aggregated into a single lakeside amenity variable based on each of these predictors having a variance inflation factor (VIF) greater than 2.5. These six significantly correlated general amenities were combined into a lake amenity score metric with a range from one to six, while boat ramps were included as a separate presence/absence variable to reflect recreation specifically for boating and fishing. Ultimately, our linear mixed-effects model included the combined built lakeside infrastructure variable, boat ramps, water quality, shoreline tree cover, and a random lake effect as predictor variables, with visitation estimates as the response variable. All statistical analyses were completed using the lme4 package in R version 4.3.1 [47].” (l. 283-301)*

Given that in some applications—such as wildlife disease monitoring or risks like building fires—dedicated VGI systems have been designed, and these can provide many advantages, I suggest the authors discuss in the Discussion section whether such a capacity might also exist in the future for monitoring lake visitation. For example, in a dedicated VGI platform, data are collected specifically to answer a targeted question, which reduces the need for extensive data preprocessing. Moreover, mechanisms for quality control (e.g., expert review or credibility scoring for active contributors) can be embedded into the system.
Some relevant examples of dedicated VGI platforms are as follows:
• Di Lorenzo, A., Zenobio, V., Cioci, D., Dall’Acqua, F., Tora, S., Iannetti, S., ... & Di Sabatino, D. (2023). A web-based geographic information system monitoring wildlife diseases in Abruzzo and Molise regions, Southern Italy. BMC Veterinary Research, 19(1), 183.
• Vahidnia, M. H., Hosseinali, F., & Shafiei, M. (2020). Crowdsource mapping of target buildings in hazard: The utilization of smartphone technologies and geographic services. Applied Geomatics, 12(1), 3–14.

*This is an excellent suggestion. We have made the following additions to our Discussion to address this comment:*

*“To address some challenges, such as disease monitoring or hazard mapping, managers have developed dedicated VGI platforms to address a specific question and minimize data processing time [69,70]. While such platforms could aid in lake visitation monitoring, this approach is dependent on recruiting and maintaining active VGI platform users. Location data derived from passive background location sharing on cellular devices rather than active posts to specific VGI sources may address some of the biases of traditional VGI data, though this cell data can be prohibitively expensive to acquire [71].” (l. 551-557)*

**Reviewer #2:**

Interesting subject, but not a new one, as there are several approaches about using VGI to study visitation patterns. The answers provided to reviewer #1 are correct and adequate to the questions raised.
My only recommendation is to update the references used, as the most recent date is from 2023.

*We thank the reviewer for their support of our manuscript and suggestion to update the references. We have added references to the following citations into the text:*

*13. Anderson LG, Rocliffe S, Haddaway NR, Dunn AM. The role of tourism and recreation in the spread of non-native species: a systematic review and meta-analysis. PloS one. 2015;10(10): e0140833.*

*36. White EM, Winder SG, Wood SA. Applying novel visitation models using diverse social media to understand recreation change after wildfire and site closure. Society & Natural Resources. 2023;36(1): 58-75.*

*49. Johnston R, Jones K, Manley D. Confounding and collinearity in regression analysis: a cautionary tale and an alternative procedure, illustrated by studies of British voting behaviour. Quality & quantity. 2018;52(4): 1957-76.*

*52. Ketchin M, Long JA. Estimating park visitation in Canadian national parks using volunteered geographic information (VGI). Journal of Outdoor Recreation and Tourism. 2025;50: 100888.*

*66. Lawson S, Monz C, Larkin A. Passive Mobile Data Analysis of Visitor Use in Parks and Protected Areas: Prospects and Challenges. Journal of Park & Recreation Administration. 2023;41(4): 95–104.*

*68. Hanson D, Wilkins EJ, Wood SH, Crowley C, Boone W, Schuster R. Monitoring recreation on federally managed lands and waters—Visitation estimation. US Geological Survey; 2025.*

*69. Di Lorenzo A, Zenobio V, Cioci D, Dall’Acqua F, Tora S, Iannetti S, ... & Di Sabatino, D. (2023). A web-based geographic information system monitoring wildlife diseases in Abruzzo and Molise regions, Southern Italy. BMC Veterinary Research, 19(1), 183.*

*70. Vahidnia MH, Hosseinali F, Shafiei M. Crowdsource mapping of target buildings in hazard: The utilization of smartphone technologies and geographic services. Applied Geomatics. 2020;12(1): 3–14.*

*71. Tsai WL, Merrill NH, Neale AC, Grupper M. Using cellular device location data to estimate visitation to public lands: Comparing device location data to US National Park Service’s visitor use statistics. Plos one. 2023;18(11): e0289922.*

*72. Merrill N, Winder SG, Hanson D, Wood SA, White E. A National Model for US Public Land Visitation. Center for Open Science. 2024.*
